# Supplementary material for: From sequence to enzyme mechanism using multi-label machine learning
Source: BMC Bioinformatics. 2014 May 19;15:150. doi: 10.1186/1471-2105-15-150 (PMC4229970; doi:10.1186/1471-2105-15-150)
Supplement: Additional file 2 — Java code of ml2db. Additional file ml2db_code.tar.gz contains the Java source code to run the multi-label machine learning experiments and save the results to database. The code’s Javadoc is included. [file 1471-2105-15-150-S2.zip › additional file 2/ml2db/ml2db/doc/main/ml2db/al/class-use/ActiveLearning.html]

Uses of Class main.ml2db.al.ActiveLearning


JavaScript is disabled on your browser.


- Overview
- Package
- Class
- Use
- Tree
- Deprecated
- Index
- Help

- Prev
- Next

- Frames
- No Frames

- All Classes

## Uses of Class main.ml2db.al.ActiveLearning

- Packages that use ActiveLearning

  | Package | Description |
  | --- | --- |
  |  |  |
  | --- | --- |
  | main.ml2db.al.learning |  |
  | test.ml2db.al |  |
  | test.ml2db.al.utility.confidence |  |
  | test.ml2db.al.utility.globaldistance |  |
  | test.ml2db.al.utility.hybrid |  |
  | test.ml2db.al.utility.neighbourhood |  |
  | test.ml2db.al.utility.pickers |  |
- - ### Uses of ActiveLearning in main.ml2db.al.learning

    Methods in main.ml2db.al.learning that return ActiveLearning

    | Modifier and Type | Method and Description |
    | --- | --- |
    |  |  |
    | --- | --- |
    | `ActiveLearning` | AlExperimenter.`getActiveLearner()` |

    Constructors in main.ml2db.al.learning with parameters of type ActiveLearning

    Constructor and Description

    `AlExperimenter(mulan.data.MultiLabelInstances trainSet,
    mulan.data.MultiLabelInstances testSet,
    DbManagers dbMan,
    MulanLearner learner,
    java.lang.String resultsDirectory,
    ActiveLearning experiment)`
  - ### Uses of ActiveLearning in test.ml2db.al

    Methods in test.ml2db.al that return ActiveLearning

    | Modifier and Type | Method and Description |
    | --- | --- |
    |  |  |
    | --- | --- |
    | `static ActiveLearning` | ActiveLearningTest.`getActiveLearningTrainsetArff()` |
    | `static ActiveLearning` | ActiveLearningTest.`getAlTestsetArffWithGlobalPositioning()` |
    | `static ActiveLearning` | ActiveLearningTest.`getAlTestsetArffWithGlobalPositioningAndConf()` |

    Methods in test.ml2db.al with parameters of type ActiveLearning

    | Modifier and Type | Method and Description |
    | --- | --- |
    |  |  |
    | --- | --- |
    | `static IdInstance` | ActiveLearningTest.`getNextStepQueryInstance(ActiveLearning activator, int step)` |
  - ### Uses of ActiveLearning in test.ml2db.al.utility.confidence

    Methods in test.ml2db.al.utility.confidence that return ActiveLearning

    | Modifier and Type | Method and Description |
    | --- | --- |
    |  |  |
    | --- | --- |
    | `static ActiveLearning` | ConfidenceMetricTest.`getALOnTestsetArffWithConfidenceMetric()` |
  - ### Uses of ActiveLearning in test.ml2db.al.utility.globaldistance

    Methods in test.ml2db.al.utility.globaldistance that return ActiveLearning

    | Modifier and Type | Method and Description |
    | --- | --- |
    |  |  |
    | --- | --- |
    | `static ActiveLearning` | GlobalDistanceMetricTest.`getAlTestsetWithGlobalPositionMetric()` |
    | `static ActiveLearning` | GlobalDistanceMetricTest.`getAlTrainsetWithGlobalPositionMetric()` |
  - ### Uses of ActiveLearning in test.ml2db.al.utility.hybrid

    Methods in test.ml2db.al.utility.hybrid that return ActiveLearning

    | Modifier and Type | Method and Description |
    | --- | --- |
    |  |  |
    | --- | --- |
    | `static ActiveLearning` | GlobalDistanceWithConfidenceMetricTest.`getAlTestsetWithGlobDistanceWithConfidenceMetric()` |
  - ### Uses of ActiveLearning in test.ml2db.al.utility.neighbourhood

    Methods in test.ml2db.al.utility.neighbourhood that return ActiveLearning

    | Modifier and Type | Method and Description |
    | --- | --- |
    |  |  |
    | --- | --- |
    | `static ActiveLearning` | NeighbourhoodExperimentDetailsTest.`getActiveLearningTestsetArffWithNN()` |
    | `static ActiveLearning` | NeighbourhoodExperimentDetailsTest.`getActiveLearningTrainsetArffWithNN()` |
  - ### Uses of ActiveLearning in test.ml2db.al.utility.pickers

    Methods in test.ml2db.al.utility.pickers that return ActiveLearning

    | Modifier and Type | Method and Description |
    | --- | --- |
    |  |  |
    | --- | --- |
    | `static ActiveLearning` | RandomAttributePickerTest.`getAlTestsetWithAttRandomPicker()` |
    | `static ActiveLearning` | AttSetByFrequencyPickerTest.`getAlTestsetWithAttSetPickerByFrequency()` |
    | `static ActiveLearning` | RandomAttributeSetPickerTest.`getAlTestsetWithAttSetRandomPicker()` |
    | `static ActiveLearning` | LabelsByFreqPickerTest.`getAlTestsetWithLabByFreqPicker()` |
    | `static ActiveLearning` | RandomAttributePickerTest.`getRandomAttPickerAL()` |

- Overview
- Package
- Class
- Use
- Tree
- Deprecated
- Index
- Help

- Prev
- Next

- Frames
- No Frames

- All Classes
